# Design Method for Combined Shear Connectors in Steel–UHPC Composite Beams

**DOI:** 10.3390/ma19081498

**Published:** 2026-04-09

**Authors:** Jingnan Ding, Tiange Gao, Jinsong Zhu

**Affiliations:** 1 School of Civil Engineering, Shandong Jianzhu University, Jinan 250101, China; dingjingnan23@sdjzu.edu.cn; 2 Key Lab of Building Structural Retrofitting and Underground Space Engineering of the Ministry of Education, Shandong Jianzhu University, Jinan 250101, China; 3 School of Civil Engineering, Tianjin University, Tianjin 300072, China; 4 Key Laboratory of Coast Civil Structure Safety of Ministry of Education, Tianjin University, Tianjin 300072, China

**Keywords:** bridge engineering, steel–UHPC composite beam, headed stud, composite dowel connector, combined connectors

## Abstract

Steel–UHPC composite beams are widely used in bridge engineering due to their high strength, durability, and suitability for prefabricated construction. However, the mechanical performance of shear connectors in UHPC differs significantly, and the uniform use of a single connector type along the beam span may result in a mismatch between connector mechanical characteristics and regional force demands, leading to suboptimal force transfer and inefficient utilization of connector capacity along the beam span. While previous studies have mainly focused on the local behavior of individual connectors, a system-level design strategy considering regional force demands is still limited. This study proposes a system-level design method for combined shear connectors in steel–UHPC composite beams, in which headed stud connectors and trapezoidal composite dowel connectors are arranged according to bending moment distribution and interface shear demand, thereby integrating connector mechanical characteristics with the spatial variation in internal forces along the beam span. The design procedure includes shear span division, longitudinal interface shear calculation, and resistance verification of different connector types. The method is applied to a practical steel–UHPC composite beam in a long-span approach bridge. Results show that headed studs provide reliable uplift resistance and ductile behavior in negative bending regions, whereas composite dowel connectors are shown to be more suitable for shear-dominated positive bending regions due to their higher shear capacity and stiffness. The combined system ensures effective composite action under different stress states and reduces total connector steel consumption compared with a stud-only layout. The proposed approach advances connector design toward performance-oriented and system-level structural optimization, providing a practical framework for connector arrangement in steel–UHPC composite beams.

## 1. Introduction

Steel-concrete composite beams have been widely adopted in bridge engineering due to their efficient structural performance, construction convenience, and favorable fatigue behavior, particularly in medium- and long-span bridges [1,2]. With the application of ultra-high performance concrete (UHPC), steel–UHPC composite beams allow for thinner bridge decks, reduced self-weight, and improved durability, making them well-suited for prefabricated and accelerated bridge construction [3,4]. To ensure effective composite action in steel–UHPC composite beams, various types of shear connectors have been proposed and extensively investigated. The most commonly studied connectors include headed stud connectors, perfobond rib (PBL) connectors, demountable mechanical connectors, and other connectors featuring mechanical interlocking mechanisms. These connectors are responsible for transferring interface shear forces and preventing relative slip or uplift between the steel girder and UHPC slab, and their mechanical performance has been shown to directly affect the stiffness, strength, and durability of composite bridge structures [5,6,7]. However, the introduction of UHPC and thin concrete slabs alters the stress state and failure mechanisms of conventional shear connectors. Existing studies indicate that different types of connectors exhibit distinct mechanical characteristics and limitations when applied in steel–UHPC composite beams, suggesting that the direct application of a single connector type may not fully satisfy the varying force demands along the beam, as different connectors exhibit distinct stiffness, strength, and deformation characteristics, which may lead to either underutilization of capacity or insufficient resistance under complex stress conditions [5].

Extensive experimental and numerical studies have shown that headed stud connectors embedded in UHPC generally exhibit higher shear capacity and stiffness than those in normal-strength concrete, with failure typically governed by stud fracture rather than concrete crushing [1,8]. To accommodate thin UHPC slabs, short and small-diameter studs are often adopted; although such studs can achieve considerable shear resistance, their slip capacity at failure is usually limited, resulting in reduced ductility and more brittle behavior [8,9], which may adversely affect deformation compatibility and crack control in negative bending regions of composite beams. In practical applications, stud connectors are commonly arranged in groups, especially in prefabricated steel–UHPC composite beams; however, pronounced group effects have been observed, leading to non-uniform force distribution and a reduction in average shear capacity and stiffness [1,3,4]. In addition, the shear behavior of stud connectors is influenced by interface configuration and construction details, as demonstrated in corrugated steel–UHPC composite bridge decks and demountable connector systems, indicating that connector performance is highly dependent on local stress conditions and detailing [6,7]. More importantly, compared with connectors featuring mechanical interlocking mechanisms, headed stud connectors exhibit relatively limited resistance to uplift and tensile separation, as their load transfer mechanism is primarily governed by shear deformation of the stud shank rather than mechanical interlocking, which may limit their effectiveness under combined shear–uplift loading conditions [5].

PBL shear connectors have been extensively investigated in steel–UHPC composite structures because of their high shear capacity, stiffness, and favorable fatigue performance. Owing to the superior compressive strength and crack resistance of UHPC, PBL connectors can effectively mobilize the bearing action of concrete dowels and transverse rebars, thereby enhancing interfacial shear resistance between steel girders and UHPC slabs [10]. Experimental studies have shown that UHPC significantly delays crack initiation and propagation around perforations, leading to stable load-slip responses and evident slip-hardening behavior in push-out and flexural tests [11,12].

The shear performance of PBL connectors in UHPC is governed by geometric and detailing parameters, including hole diameter, plate thickness, transverse rebar diameter, embedment depth, and end-bearing conditions. In particular, deeply embedded PBL connectors can activate end-bearing resistance and markedly increase shear strength [13,14]. Similarly to stud connectors, group effects have also been observed for PBLs: increasing the number of perforations or reducing spacing tends to decrease the average shear resistance per hole because of stress interaction among concrete dowels [15,16]. To improve prediction accuracy, analytical and data-driven models have been developed, with recent interpretable machine-learning approaches showing superior performance for UHPC and multi-hole configurations, although they remain dependent on shear-dominated test data [17].

**Figure 1:**
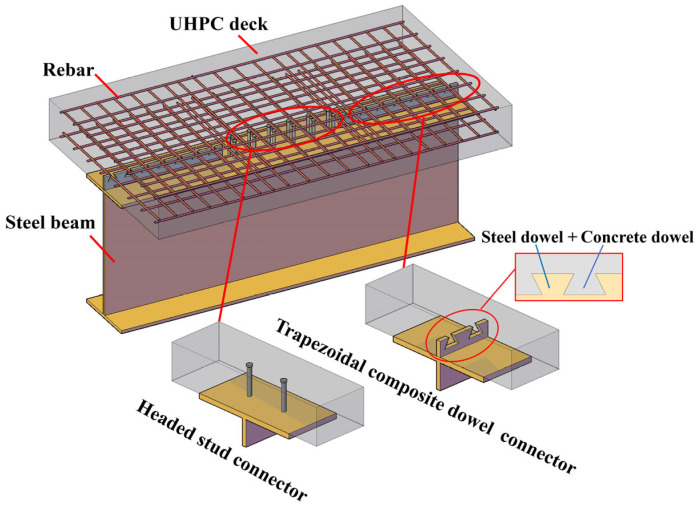
Steel–UHPC composite beam with combined shear connectors.

Compared with headed studs, PBL connectors primarily resist interface shear through concrete dowel action, transverse rebar tension, and steel plate bearing, resulting in high shear capacity but limited vertical deformation and uplift resistance [5]. Therefore, the exclusive use of PBL connectors may not fully satisfy the combined shear and uplift demands in steel–UHPC composite beams under varying stress states. The composite dowel connector, a semi-open shear connector derived from the PBL connector (see Figure 1), has been proposed. It consists of a steel dowel and a concrete dowel working together, in which shear resistance is mobilized through concrete dowel action, transverse reinforcement tension, and steel bearing. This mechanism results in higher shear capacity and stiffness compared with headed studs and enhances performance under combined shear–uplift loading conditions.

For steel–UHPC composite bridges, the current design specifications, such as the Code for Design of Steel-Concrete Composite Bridges (GB 50917-2013), recommend that shear connectors should be uniformly distributed within the corresponding force-resisting regions using the same connector type [18]. Substantial research has been conducted on individual shear connector types in steel–UHPC composite beams; most studies focus on isolated push-out behavior or local structural response, with limited consideration of system-level connector design under realistic bridge loading conditions. In continuous composite beams, the interface is subjected to spatially varying combinations of shear, bending, and uplift actions. Under such complex stress states, uniform use of a single connector type may lead to potentially conservative design in some regions and insufficient performance in others, depending on the local stress state and connector characteristics [19,20,21]. Therefore, a rational connector configuration strategy that integrates the distinct mechanical characteristics of different connectors within a unified structural scheme remains needed.

The authors have previously carried out systematic experimental and numerical investigations on headed stud connectors and composite dowel connectors in steel–UHPC composite beams [1,22,23], providing a solid mechanical foundation for structural-level application. Building upon these findings, the present study proposes a combined connector system in which different connector types are arranged according to regional force demands along the beam span (Figure 1). The present study aims to bridge the gap between connector-level mechanical studies and structural-level design methodology by explicitly linking connector mechanical characteristics with the spatial distribution of internal forces along the beam span. The proposed approach establishes a practical design framework in which shear span regions are determined based on bending moment distribution, the longitudinal interface shear force is evaluated according to composite beam theory, and the resistance of different connector types is verified using relevant bridge design provisions, including the Code for Design of Steel-Concrete Composite Bridges (GB 50917-2013) [18] and the Specifications for Design of Highway Steel Bridges (JTG D64-2015) [24]. The method is further implemented through a long-span steel–UHPC composite bridge, in which structural internal forces are obtained from a finite element model and used to determine the connector arrangement along the beam span. By coordinating connector characteristics with regional stress requirements, the proposed approach advances existing research from single-connector evaluation toward performance-oriented structural design of steel–UHPC composite bridges.

## 2. Design of Combined Shear Connectors

### 2.1. Division of Shear Spans and Calculation of Longitudinal Shear Force

**Figure 2:**
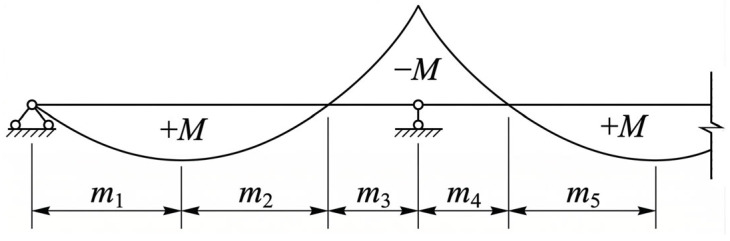
Division of shear span regions for a continuous composite beam.

To fully utilize the respective mechanical advantages of headed stud connectors and composite dowel connectors, the shear span regions of steel-UHPC composite beam bridges are determined prior to the arrangement of the combined connectors. According to Section 7.5.2 of the Chinese Code for Design of Steel-Concrete Composite Bridges (GB 50917-2013) [18], the shear span regions of continuous composite beams are divided based on the locations of maximum bending moments and zero-moment points (Figure 2), which provides a practical basis for shear span partitioning in engineering design. As shown in Figure 2, shear spans *m*_1_ and *m*_2_ are combined, and trapezoidal composite dowel connectors are arranged uniformly within the combined positive moment regions (*m*_1_, *m*_2_ and *m*_5_). Meanwhile, shear spans *m*_3_ and *m*_4_ are merged and headed stud connectors are uniformly arranged within the combined negative moment region. In each shear span region, only one type of shear connector is adopted to ensure construction convenience and clarity of force transfer, thereby avoiding the additional detailing complexity and force-transfer uncertainty that may arise from locally mixed connector layouts.

According to Section 11.4.3 of the Specifications for Design of Highway Steel Bridges (JTG D64-2015) [24], the longitudinal horizontal shear force along the steel–concrete interface is calculated based on uncracked section analysis, as given in Equation (1).

(1) $$V_{ld}=\frac{V_{d}S}{I_{un}}$$

where *V*_d_ is the longitudinal shear force per unit length along the steel-concrete interface, *V*_d_ is the vertical shear force of the composite beam section, *S* is the moment of area of the concrete slab about the neutral axis of the composite section, and *I*_un_ is the moment of inertia of the uncracked composite section.

### 2.2. Design of Headed Stud Connectors

Design formulas for the shear resistance of headed stud connectors in steel-concrete composite bridges are provided in the Chinese code GB 50917-2013 [18], the American AASHTO LRFD Bridge Design Specifications [25], and the European Eurocode 4 [26]. However, previous studies have indicated that, although these design formulas incorporate safety factors, their direct application to steel–UHPC composite beams may lead to relatively conservative predictions in some cases, with the calculated shear resistance being noticeably lower than the corresponding experimental results [4,27]. Based on existing literature and experimental results for steel–UHPC headed stud connectors, Wu [28] proposed a shear resistance calculation formula for headed stud connectors embedded in UHPC. The formula was developed on the basis of experimental studies on headed stud connectors in UHPC and is adopted here for the shear resistance verification of headed studs in the present design framework. The ultimate shear resistance *V*_nu_ can be calculated using Equations (2) and (3):

(2) $$V_{nu}=\varphi A_{s}f_{uta}/\lambda$$

(3) $$\varphi =0.85+f_{c}/f_{uta}$$

where *A*_s_ is the cross-sectional area of the stud shank, *f*_uta_ is the ultimate tensile strength of the stud, *f*_c_ is the compressive strength of UHPC, and *λ* is the partial safety factor for resistance. According to Wu [28], the value of *λ* was obtained by regression analysis of experimental data, with a fitted value of 0.94 indicating good agreement between calculated and test results. For design purposes, a more conservative value of *λ* = 1.24 is recommended, which is adopted in the present study.

For the negative moment region, the shear resistance verification of headed stud connectors should satisfy Equation (4), which is based on the design provisions for steel-concrete composite bridges specified in the Chinese Code for Design of Steel-Concrete Composite Bridges (GB 50917-2013) [18]:

(4) $$V_{ld}\leq n_{1}V_{nu}$$

where *n*_1_ is the number of headed stud connectors arranged per meter.

The ultimate uplift resistance *T* of headed stud connectors can be calculated using Equation (5), which was developed based on the authors’ previous experimental and numerical studies on the uplift behavior of headed stud connectors in UHPC [22,23], considering the corresponding failure mechanisms:

(5) $$T=\min\left(f_{t}\frac{\pi \left[{\left(d_{h}+2h_{s}\tan\beta \right)}^{2}-d_{s}^{2}\right]}{4},\frac{\pi d_{s}^{2}}{4}f_{uta}\right)$$

where *f*_t_ is the tensile strength of UHPC, *d*_h_ is the diameter of the stud head, *d*_s_ is the diameter of the stud shank, *h*_s_ is the length of the stud shank, and *β* is the angle between the punching failure surface and the vertical plane, taken as 43°.

The uplift resistance verification of headed stud connectors in the negative moment region should satisfy Equation (6):

(6) $$T_{d}\leq n_{2}T$$

where *T*_d_ is the maximum uplift force between steel and UHPC in the composite beam section under negative bending, obtained from simplified structural analysis of the composite beam based on sectional force equilibrium, and *n*_2_ is the total number of headed stud connectors arranged within the composite beam section.

### 2.3. Design of Trapezoidal Composite Dowel Connectors

Trapezoidal composite dowel connectors are arranged in the positive moment regions of steel–UHPC composite beams. The authors have also conducted extensive experimental and numerical studies on the shear behavior of trapezoidal composite dowel connectors embedded in UHPC [10,23]. Based on the test and numerical results, the governing failure modes of trapezoidal composite dowel connectors mainly include steel dowel root failure and UHPC dowel root failure. The shear resistance model was therefore established by combining the resistance contributions corresponding to these two failure modes. The ultimate shear resistance *V*_cu_ of trapezoidal composite dowel connectors can be calculated using Equation (7):

(7) $$V_{cu}=\min(\frac{tb_{s}^{2}f_{y}}{\lambda \sqrt{4h^{2}+3b_{s}^{2}}},\eta e_{x}^{2}f_{ct}(1+\frac{E_{r}A_{r}}{E_{c}A_{c}}))$$

where *b*_s_ is the short side length of the trapezoidal steel rib, *f*_y_ is the yield strength of steel, *h* is the height of the trapezoidal rib, *η* is the modification coefficient accounting for the contribution of the concrete dowel (taken as 2), *e*_x_ is the spacing between adjacent steel ribs, *E*_c_ is the elastic modulus of UHPC, *E*_r_ is the elastic modulus of reinforcing bars, *A*_r_ is the cross-sectional area of reinforcing bars passing through the UHPC dowel, and *A*_c_ is the area of the UHPC dowel.

For the positive moment region, the shear resistance verification of trapezoidal composite dowel connectors should satisfy Equation (8), in which the design verification is performed by introducing the partial safety factor for resistance into the shear resistance model given in Equation (7):

(8) $$V_{cu}=\min(\frac{tb_{s}^{2}f_{y}}{\lambda \sqrt{4h^{2}+3b_{s}^{2}}},\eta e_{x}^{2}f_{ct}(1+\frac{E_{r}A_{r}}{E_{c}A_{c}})/\lambda )$$

where *n*_3_ is the actual number of trapezoidal composite dowel connectors arranged per meter, and λ is the partial safety factor for resistance, taken as 1.1.

## 3. Combined Connector Design Application

### 3.1. Project Description

**Figure 3:**
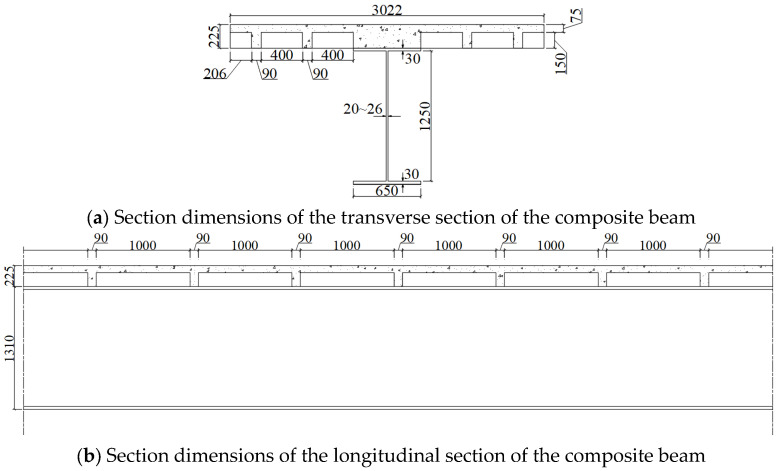
The dimensions of the steel–UHPC waffle slab composite beam (unit: mm).

A long-span approach bridge was selected as the engineering background to conduct the combined connector design for a steel–UHPC composite bridge. The superstructure consists of a steel I-beam with a vertical web and a UHPC waffle deck slab. The total depth of the composite beam is 1535 mm, including a steel beam depth of 1310 mm, with a beam spacing of 3022 mm. The web height (*h*_sw_) is 1250 mm, and the web thickness (*t*_sw_) varies from 20 mm in the positive bending region to 26 mm in the negative bending region. The flange width (*b*_sf_) and thickness (*t*_sf_) are 650 mm and 30 mm, respectively. The UHPC waffle slab has a panel thickness of 75 mm. The longitudinal and transverse rib widths are both 90 mm, and the rib height is 150 mm. The spacing of longitudinal ribs is 400 mm, while that of transverse ribs is 1000 mm. The dimensions of the steel–UHPC waffle slab composite beam are shown in Figure 3.

The precast waffle slab was fabricated using UHPC with a measured cube compressive strength of 127 MPa. In the connector resistance calculations, the parameter *f*_c_ represents the design value of the concrete compressive strength, which is taken as 87.6 MPa after applying a conversion factor of 1.45, which is based on experimental observations and reflects the conversion from measured cube compressive strength to design compressive strength by incorporating safety margins in structural design. The tensile strength *f*_t_ and elastic modulus *E*_c_ of UHPC were taken as 8.27 MPa and 50 GPa, respectively. The steel beam was made of Q420q steel with a yield strength (*f*_y_) of 335 MPa. HRB400 reinforcement was adopted for the waffle slab, with an elastic modulus (*E*_s_) of 2.0 × 10^5^ MPa.

### 3.2. Combined Connector Design

**Figure 4:**
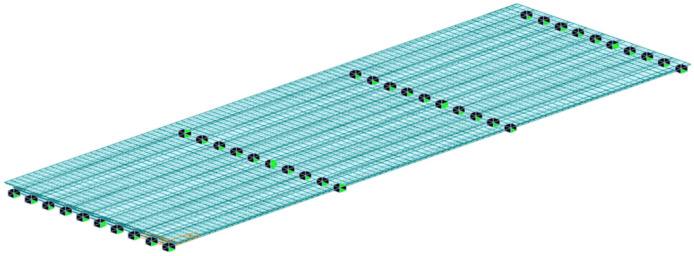
Calculation model of the approach bridge of a certain long bridge.

A three-dimensional finite element model was established using Midas Civil to obtain the internal force distribution of the composite beam under the design load combinations. The steel beam and the UHPC waffle slab were simulated using beam elements, forming a double-beam element model to represent the composite structural behavior. In the numerical model, the interaction between the steel beam and the UHPC slab was simplified as a rigid connection, assuming full composite action between the two components, which is consistent with the design stage where sufficient shear connectors are provided to achieve composite behavior. Under this assumption, the calculated shear flow represents the design demand along the interface, and the influence of interface slip is not explicitly considered. In addition, the connections between the steel girder and the supports were also modeled as rigid constraints in order to represent the boundary conditions of the bridge structure, which is a common simplification in structural design for determining the internal force distribution of composite beams. In this study, the primary objective is to evaluate the distribution of internal forces for connector design, and the influence of support flexibility on the global force distribution is considered secondary and therefore not explicitly included. The global model consisted of 2745 beam elements and 1860 nodes. The specific calculation model is shown in Figure 4.

**Figure 5:**
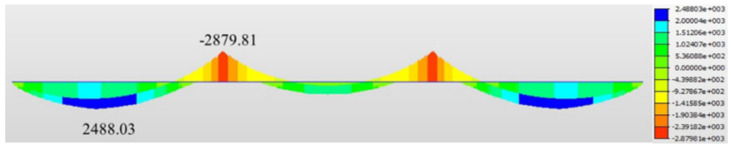
The bending moment envelope of steel–UHPC waffle slab composite beam (unit: kN·m).

**Figure 6:**
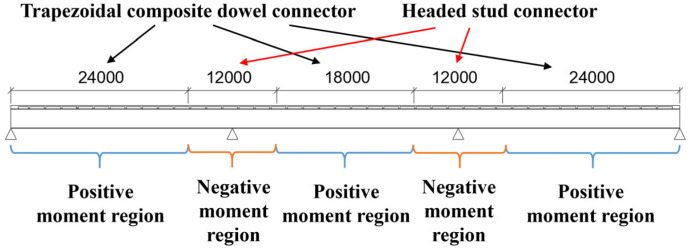
The shear span division and connector layout (unit: mm).

Based on the analysis results obtained from the Midas Civil model, the bridge was divided into shear span regions. The bending moment envelope of the steel–UHPC waffle slab composite beam under the basic load combination is shown in Figure 5. Using the zero-moment locations as boundaries, the entire bridge was divided into five segments. Trapezoidal composite dowel connectors were uniformly arranged in the three positive moment regions, while headed stud connectors were uniformly arranged in the two negative moment regions. The shear span division and connector layout of the steel–UHPC waffle slab composite beam are illustrated in Figure 6. The regions within 6 m from the two interior supports along the longitudinal bridge direction were defined as negative moment regions, where the distance of 6 m corresponds to the locations of zero bending moments obtained from the Midas Civil analysis results (Figure 5).

In the negative moment region, headed stud connectors were adopted between the concrete slab and steel beam. The headed stud was 22 mm in diameter and 180 mm in height, and was made of ML15 with an ultimate tensile strength (*f*_uta_) of 400 MPa. Twelve-headed studs per meter were arranged, with longitudinal spacing of 165 mm and transverse spacing of 100 mm, which satisfy the spacing requirements specified in relevant design codes [18]. The ultimate shear capacity of a single-headed stud was calculated using Equation (2), resulting in *V*_nu_ = 32.75 kN.

The longitudinal interface shear force per unit length was calculated using Equation (1), resulting in *V*_ld_ = 1145 kN. The shear resistance in the negative moment region was verified using Equation (4) as follows, and the requirements were satisfied.

(9) $$V_{ld}=1145\;\mathrm{kN}\leq n_{1}V_{nu}=1179\;\mathrm{kN}$$

The uplift resistance of the headed stud connectors was calculated using Equation (5), and the result was shown in Equation (10). The results indicate that the uplift resistance is governed by the tensile capacity of the stud shank. Pull-out failure corresponds to stud shank fracture, which can be regarded as a ductile failure mode based on the material behavior of steel, and is consistent with structural design requirements. The uplift resistance was further verified using Equation (6), confirming compliance with design requirements (Equation (12)).

(10) $$\begin{array}{l} T=\min\left(8.27\times \frac{\pi \left[{\left(32+2\times 170\times \tan43{}^{\circ}\right)}^{2}-22^{2}\right]}{4},\frac{\pi \times 22^{2}}{4}\times 400\right)\\ =152.1\;\mathrm{kN} \end{array}$$

(11) $$T_{d}=128\;\mathrm{kN}\leq n_{2}T=304\;\mathrm{kN}$$

In the positive moment region, trapezoidal composite dowel connectors were adopted. The design parameters are listed in Table 1, and six connectors per meter were arranged.

The ultimate shear capacity of the trapezoidal composite dowel connectors was calculated using Equation (7), and the result was shown in Equation (12). The results indicate that the shear capacity is governed by the ultimate shear resistance of the steel dowel. Longitudinal push-out failure is characterized by root failure of the steel dowel, as identified from the shear resistance model established based on previous experimental and numerical studies, which is a ductile failure mode and satisfies structural requirements.

(12) $$\begin{array}{l} V_{cu}=\min(\frac{25\times 70^{2}\times 335}{1.1\sqrt{4\times 55^{2}+3\times 70^{2}}},2\times 170^{2}\times 8.27\times (1+\frac{2\times 10^{5}\times 380}{5\times 10^{4}\times 3850})/1.1)\\ =227.9\;\mathrm{kN} \end{array}$$

The shear resistance was verified using Equation (8), confirming that the design requirements were satisfied (Equation(13)).

(13) $$V_{ld}=1145\;\mathrm{kN}\leq n_{1}V_{nu}=1367\;\mathrm{kN}$$

### 3.3. Optimization Comparison of Connector Arrangement

In the preliminary design, headed stud connectors were adopted and uniformly distributed along the entire bridge. To assess the effectiveness of the proposed combined connector arrangement, a comparative analysis was conducted against this conventional stud-only layout. As summarized in Table 2, although the trapezoidal composite dowel connector has a higher steel consumption per connector in the positive moment region, its substantially higher shear capacity allows the required number of connectors per meter to be reduced (from 12 to 6). Consequently, the steel consumption of connectors per meter in the positive moment region is reduced from 6.84 kg/m to 6.48 kg/m, while the negative moment region remains unchanged (6.84 kg/m) because headed studs are used in both layouts. Overall, the total connector steel consumption of the bridge is reduced from 615.74 kg (stud-only) to 591.63 kg (combined layout), demonstrating improved material utilization efficiency.

From a structural mechanics perspective, the positive moment regions of continuous composite beams are primarily governed by interface shear demand, where connector shear capacity and stiffness dominate the performance of composite action. In contrast, negative moment regions are more susceptible to uplift and tensile separation between the steel girder and UHPC slab, where stable uplift resistance and deformation compatibility become critical. Accordingly, the proposed combined connector arrangement is not merely a reduction in connector quantity, but a redistribution of connector resistance in accordance with the governing mechanical demands in different regions of the beam. By assigning different connector types to regions with distinct mechanical requirements, the proposed strategy enables a more efficient utilization of connector resistance along the beam span. Beyond the reduction in steel consumption, the combined layout enhances the reliability of composite action by avoiding over-reinforcement in shear-dominated regions and potential underperformance in uplift-sensitive zones. This comparison indicates that the proposed arrangement provides both mechanical rationality and economic benefits, offering a practical and performance-oriented alternative for connector design in steel–UHPC composite girder bridges.

## 4. Conclusions

(1) A performance-oriented combined connector design approach for steel–UHPC composite beams is proposed, in which headed stud connectors and trapezoidal composite dowel connectors are arranged according to regional force demands along the beam span. The approach provides a structured framework for integrating shear span division, interface shear evaluation, and connector resistance verification in a design-oriented manner.
(2) The mechanical characteristics of different connectors were rationally matched with regional structural demands. Headed stud connectors provided reliable uplift resistance and ductile failure behavior in negative moment regions, while trapezoidal composite dowel connectors offered high shear resistance and stiffness in positive moment regions, ensuring coordinated composite action under varying stress states.
(3) Application to a practical steel–UHPC composite bridge demonstrated that the proposed approach can satisfy shear and uplift resistance requirements and ensures reliable composite action under service and ultimate limit states. The study provides an extension from single-connector evaluation toward a more integrated design-oriented framework. However, the current work is limited to a design-stage assessment based on a single case study, and further validation through experimental and parametric investigations is needed.
(4) Compared with the conventional headed stud-only layout, the combined connector scheme shows a modest improvement in material utilization efficiency and a reduction in connector steel consumption. Although the reduction ratio is relatively small at the unit-length level, its cumulative effect at the structural scale may still be meaningful. The proposed approach offers a practical basis for more refined and performance-based design of steel–UHPC composite bridge structures.

## Figures and Tables

**Table 1:** Design parameters of trapezoidal composite dowel connectors for steel–UHPC waffle slab composite beam.

Connector	*h*/mm	*a*_s_/mm	*b*_s_/mm	*a*_c_/mm	*b*_c_/mm	*t*/mm
trapezoidal composite dowel connector	55	130	70	100	40	25

Note: *h* is the height of the steel dowel, *a*_s_ and *b*_s_ represents the long side and short side of the trapezoidal steel dowel, respectively, *a*_c_ and *b*_c_ represent the long side and short side of the concrete dowel, respectively, and *t* represents the thickness of the steel dowel.

**Table 2:** The comparison of steel consumption between the headed stud-only layout and the combined connectors layout.

Connectors Arrangement	Headed Stud-Only Layout	Combined Connectors Layout
Steel consumption per connector in the positive moment region/kg	0.57	1.08
Number of connectors per meter in the positive moment region	12	6
Steel consumption of connectors per meter in the positive moment region/kg	6.84	6.48
Steel consumption per connector in the negative moment region/kg	0.57	0.57
Number of connectors per meter in the negative moment region	12	12
Steel consumption of connectors per meter in the negative moment region/kg	6.84	6.84
Total connector steel consumption of the bridge/kg	615.74	591.63

## Data Availability

The original contributions presented in this study are included in the article. Further inquiries can be directed to the corresponding author.
